# Relative Energy Deficiency in Sport (REDs) in Ultra-Trail Running: Implications for Performance, Injury, and Long-Term Health

**DOI:** 10.7759/cureus.111971

**Published:** 2026-07-02

**Authors:** Rachael Cullinan

**Affiliations:** 1 Orthopaedics, Golden Jubilee National Hospital, Glasgow, GBR

**Keywords:** athlete health, athlete health and performance, endurance running, endurance sport, low energy availability, reds, relative energy deficiency in sport, sports nutrition, trail running, ultra marathon

## Abstract

Ultra-trail running has experienced substantial global growth, yet the nutritional and physiological consequences of prolonged participation remain incompletely understood. Relative energy deficiency in sport (REDs), resulting from problematic low energy availability (LEA), has emerged as an important concern within endurance sports; however, evidence specific to ultra-trail athletes remains fragmented. This narrative review synthesises current evidence relating to the development, manifestations, and consequences of REDs in ultra-trail running. Available literature suggests that the combination of high training volumes, prolonged competition, inadequate energy intake, gastrointestinal limitations, sleep disruption, and environmental stressors may place ultra-trail athletes at heightened risk of chronic LEA. Consequences extend beyond impaired performance and recovery to include endocrine dysfunction, compromised bone health, increased injury susceptibility, immune disturbance, and adverse psychological outcomes. Despite growing recognition of REDs within endurance sport, prevalence estimates in ultra-trail populations remain poorly defined, and validated sport-specific screening and management strategies are lacking. Greater awareness among athletes, coaches, and healthcare professionals is required to facilitate earlier recognition and intervention. Future research should prioritise prospective studies, sport-specific diagnostic approaches, and targeted prevention strategies to better support athlete health, performance, and long-term participation in ultra-trail running.

## Introduction and background

Participation in endurance running has increased substantially during recent decades, accompanied by rapid expansion of ultramarathon and ultra-trail running events worldwide. Once regarded as a niche activity pursued by a small number of endurance enthusiasts, ultra-trail running has evolved into a globally recognised competitive sport attracting participants across a broad spectrum of age groups and performance levels [1,2]. The popularity of internationally recognised events such as the Ultra-Trail du Mont-Blanc (UTMB), Western States Endurance Run, Hardrock 100, and increasingly long-distance races exceeding 200 miles has contributed to unprecedented growth within the discipline [1,2].

The physiological demands of ultra-trail running differ substantially from those encountered in traditional road racing. Athletes are required to negotiate prolonged periods of exercise over mountainous and technically challenging terrain while simultaneously managing nutrition, hydration, thermoregulation, sleep deprivation, and recovery [3]. Successful completion of ultra-trail events depends upon complex interactions between aerobic fitness, musculoskeletal resilience, nutritional status, pacing strategy, psychological adaptability, and environmental tolerance [3].

Despite these challenges, participation is often associated with significant physical and psychological benefits. Ultra-trail runners frequently report enhanced wellbeing, resilience, social connectedness, and personal achievement [4]. However, the same factors that contribute to performance may also increase susceptibility to chronic physiological stress. In particular, the substantial energy demands imposed by high training volumes and prolonged competition can create conditions in which energy intake fails to meet energy expenditure [5].

Low energy availability (LEA) occurs when dietary energy intake is insufficient to support both exercise expenditure and the physiological requirements necessary to maintain health [6]. When sustained, LEA is the primary physiological driver of relative energy deficiency in sport (REDs), a syndrome encompassing widespread physiological and psychological dysfunction. Energy availability represents the amount of dietary energy remaining for normal physiological function after accounting for exercise energy expenditure and is commonly expressed as kilocalories per kilogram of fat-free mass per day (kcal/kg FFM/day). When energy intake is insufficient to meet the combined demands of exercise and essential biological processes, the body initiates adaptive responses aimed at conserving energy and may result in REDs [5,7]. The International Olympic Committee (IOC) originally introduced the REDs framework in 2014 to expand upon the concept of the female athlete triad and recognise the broader systemic consequences of inadequate energy availability [5,7,8]. Subsequent consensus updates have further refined understanding of REDs and acknowledged their occurrence in both male and female athletes [5].

Relative energy deficiency in sport affects multiple organ systems, including endocrine, reproductive, skeletal, immune, cardiovascular, metabolic, gastrointestinal, and psychological domains [5,9]. Consequences may include menstrual dysfunction, impaired bone health, reduced testosterone concentrations, recurrent illness, mood disturbance, impaired recovery, and diminished athletic performance [5,10]. Importantly, these manifestations often develop gradually and may remain unrecognised for prolonged periods.

Although REDs has received increasing attention within sports medicine literature, research has largely focused upon sports traditionally associated with weight sensitivity or aesthetic performance, such as gymnastics, ballet, figure skating, and distance running [11,12]. Comparatively little attention has been directed toward ultra-trail running despite several features suggesting heightened vulnerability. Athletes routinely undertake training volumes exceeding 10 to 20 hours per week, participate in events associated with extreme energy expenditure, and frequently encounter barriers to adequate fuelling, including appetite suppression and gastrointestinal dysfunction [5,8]. Furthermore, endurance sport culture often normalises fatigue, weight loss, and high training loads, potentially obscuring early warning signs of LEA and REDs [13].

The purpose of this narrative review is therefore to examine the current understanding of REDs within ultra-trail running. Particular emphasis is placed upon defining the unique characteristics of the sport, exploring mechanisms contributing to LEA, evaluating implications for performance and injury, and identifying future research priorities. Through improved awareness and understanding, clinicians, coaches, and athletes may be better equipped to recognise and address REDs before substantial health and performance consequences occur.

Defining ultra-trail running and its physiological demands

A clear definition of ultra-trail running is essential because substantial heterogeneity exists across endurance running disciplines. The International Trail Running Association (ITRA) defines trail running as running conducted primarily on natural terrain with limited sections of paved surfaces [14]. Ultra-trail running represents a subset of trail running in which race distance exceeds the traditional marathon distance of 42.195 km [14]. In practice, ultra-trail events vary considerably in both distance and duration. Common race formats include 50 km, 80 km, 100 km, 100-mile, 200-mile, and multi-day stage events, although races exceeding 300 km have become increasingly common [1,2]. Unlike road ultramarathons, ultra-trail events typically incorporate substantial cumulative elevation gain, technical terrain, variable weather conditions, and logistical challenges relating to nutrition, hydration, and equipment management. The physiological demands of ultra-trail running extend beyond those associated with exercise duration alone [3,15,16]. Elevation changes require repeated transitions between concentric and eccentric muscular loading, increasing neuromuscular fatigue and musculoskeletal stress [17,18]. Technical terrain necessitates constant adjustments in balance and proprioception, while environmental conditions may expose athletes to extremes of heat, cold, altitude, and precipitation [17,18]. Furthermore, events frequently continue overnight, introducing sleep deprivation as an additional physiological stressor [17-19]. Energy expenditure during ultra-trail running is highly variable but may substantially exceed that observed during conventional endurance events. Depending upon body mass, terrain, environmental conditions, and race duration, athletes may expend several thousand kilocalories during a single competition [3,16,20]. Multi-day races may result in cumulative energy deficits that are impossible to replace fully despite optimal nutritional planning and can lead to frequent gastrointestinal distress [16,20-22].

The demographic profile of ultra-trail runners has also evolved. Earlier perceptions of the sport as predominantly male have become less accurate as female participation continues to increase globally [1]. Furthermore, participants now encompass a broad range of competitive abilities, from elite athletes competing for international rankings to recreational runners seeking personal achievement and adventure.

Importantly, ultra-trail running culture often emphasises self-sufficiency, perseverance, and tolerance of discomfort. While these characteristics contribute positively to performance and community identity, they may inadvertently encourage athletes to normalise chronic fatigue, persistent injury, disrupted menstrual function, and inadequate recovery [3,15,23,24]. Consequently, symptoms potentially indicative of REDs may remain unrecognised or be interpreted as expected consequences of training [24].

From a sports medicine perspective, ultra-trail running, therefore, represents a unique environment in which substantial physiological stress, prolonged energy expenditure, nutritional challenges, and cultural influences converge [3,15,16,20,24]. These characteristics distinguish the sport from both conventional road running and shorter endurance disciplines and provide a strong theoretical basis for increased susceptibility to LEA and REDs.

## Review

Methods 

This narrative review was undertaken using a structured literature search to identify evidence relating to REDs, LEA, and ultra-trail running. Searches were conducted in PubMed, Scopus, and Google Scholar in March 2026 using combinations of the terms “Relative Energy Deficiency in Sport”, “RED-S”, “REDs”, “low energy availability”, “energy deficiency”, “ultramarathon”, “ultra-trail running”, “trail running”, “endurance athlete”, “female athlete triad”, “bone stress injury”, “sports nutrition”, and “exercise endocrinology”. The date range used was 2005 to 2026. 

Eligible publications included original research studies, systematic reviews, meta-analyses, narrative reviews, consensus statements, and position stands published in English that examined LEA, REDs, nutritional physiology, endocrine function, bone health, injury risk, performance, or athlete health within endurance and ultra-endurance sports. Given the limited volume of literature specific to ultra-trail running, evidence from related endurance disciplines was included where findings were considered relevant to the physiological demands of ultra-trail athletes. Studies focusing exclusively on non-athletic populations, paediatric populations, or conditions unrelated to energy availability were excluded.

Titles and abstracts were screened for relevance, followed by full-text assessment of potentially eligible articles. Additional relevant publications were identified through manual review of reference lists from key studies and IOC consensus statements. Evidence was synthesised narratively owing to heterogeneity in study populations, methodologies, outcome measures, and definitions of LEA and REDs.

As this is a narrative review rather than a systematic review, a formal risk-of-bias assessment was not performed. However, priority was given to higher-level evidence, including systematic reviews, meta-analyses, prospective cohort studies, and international consensus statements. Study limitations and applicability to ultra-trail running were considered throughout the interpretation of findings.

Current evidence for REDs in endurance and ultra-endurance athletes

Understanding the epidemiology of REDs within ultra-trail running remains challenging because relatively few studies have specifically examined this population. Much of the available evidence originates from broader endurance sport cohorts, including distance runners, cyclists, triathletes, and military personnel [21,22,25-31]. Consequently, current understanding of REDs in ultra-trail athletes is derived largely from related endurance populations rather than direct, sport-specific investigation [11,32].

Determining the true prevalence of REDs is inherently difficult. It represents a syndrome rather than a single disease entity, with manifestations varying considerably between individuals and sporting disciplines [11,32]. Furthermore, diagnostic criteria continue to evolve following successive IOC consensus statements, while many athletes may exhibit subclinical manifestations of LEA without developing overt physiological dysfunction [11,32-34]. As a result, prevalence estimates vary substantially depending on the population studied and the assessment methods employed.

Low energy availability appears to be common across endurance sports. Studies involving competitive distance runners have reported prevalence estimates ranging from approximately 22% to more than 60%, depending upon sex, competition level, and assessment methodology [11,12,34-36]. Female endurance athletes consistently demonstrate higher rates of LEA than non-athletic controls, although increasing evidence suggests that male athletes may also be substantially affected [11,32,37-39].

Epidemiological studies frequently utilise screening tools such as the Low Energy Availability in Females Questionnaire (LEAF-Q), which assesses menstrual function, gastrointestinal symptoms, and injury history [40]. These investigations have demonstrated that a considerable proportion of female endurance athletes exhibit features associated with increased REDs risk, particularly menstrual dysfunction and susceptibility to bone stress injury [13,32,36,41]. However, prevalence estimates should be interpreted with caution. Reported rates vary substantially according to the population studied, the definition of LEA employed, and the screening methodology utilised. Questionnaire-based approaches identify athletes at risk of LEA rather than directly measuring energy availability, while laboratory-based assessments are resource-intensive and difficult to perform in large athletic populations [9,32,42].

Accurate measurement of energy availability remains challenging in free-living athletes because it requires detailed quantification of both energy intake and exercise energy expenditure. Consequently, many studies rely upon surrogate markers, including hormonal disturbances, menstrual dysfunction, impaired bone health, recurrent injury, or questionnaire-derived risk scores [9,32,42]. These methodological limitations contribute to uncertainty regarding the true prevalence of LEA and REDs across endurance sports.

Data specific to ultra-trail running remain particularly limited. The prolonged duration of training and competition, high cumulative energy expenditure, environmental stressors, and logistical barriers to adequate fuelling suggest that ultra-trail athletes may be at especially high risk of chronic LEA. However, few studies have specifically examined the prevalence of REDs in ultra-trail populations, and existing investigations are often limited by small sample sizes, cross-sectional designs, and reliance on screening questionnaires rather than direct physiological assessment. As a result, the epidemiology of REDs in ultra-trail running remains incompletely characterised despite strong theoretical and physiological justification for concern.

From the Female Athlete Triad to REDs

Much of the early literature examining energy deficiency in athletes focused on the female athlete triad, comprising LEA, menstrual dysfunction, and impaired bone health [11]. Studies involving female endurance athletes consistently demonstrated associations between menstrual irregularities, reduced bone mineral density, and increased risk of stress fracture [13,32,35,43]. These findings formed the foundation for the development of the broader REDs framework, which recognises that inadequate energy availability affects multiple physiological systems beyond reproductive and skeletal health [7,8,11,24]. Importantly, the transition from the female athlete triad to REDs has broadened recognition that male athletes may also experience clinically significant consequences of chronic LEA. This shift is particularly relevant to ultra-endurance sport, where male participation remains high and traditional assumptions may contribute to under-recognition of REDs among men [34,37,39,44].

REDs in Male Endurance Athletes

Although REDs research has historically focused on female athletes, growing evidence demonstrates that male endurance athletes are also vulnerable to chronic LEA and its physiological consequences [34,37-39]. Reduced testosterone concentrations, impaired hypothalamic-pituitary-gonadal axis function, diminished libido, impaired recovery, and reduced bone health have all been reported in endurance-trained men [34,39,44,45]. The proposed exercise hypogonadal male condition (EHMC) has generated particular interest, although the extent to which it represents physiological adaptation, chronic LEA, or a combination of both remains debated [38,39,44].

Diagnosis in male athletes remains challenging because there is no readily identifiable physiological marker equivalent to menstrual dysfunction in females that can be determined on a generalised history taking. However, the lowest 25% of total or free testosterone, total or free triiodothyronine (T3), can be used as markers, as recommended by the IOC REDs Clinical Assessment Tool version 2 (CAT2). Yet, these tests are expensive and not readily available throughout primary care [8]. Consequently, REDs may remain undetected until athletes present with recurrent injury, unexplained fatigue, impaired recovery, or declining performance [24,32,46].

Evidence in Trail and Ultra-Endurance Athletes

Direct evidence relating specifically to trail runners and ultramarathon athletes remains comparatively limited. Nevertheless, the available literature suggests that factors associated with LEA and REDs may be highly prevalent within these populations [24,31,47-49]. Studies involving trail and ultra-endurance athletes have reported menstrual dysfunction, low body mass index, disordered eating behaviours, and nutritional intakes that appear insufficient relative to training demands [15,20,47-49]. Race nutrition studies consistently demonstrate that athletes consume substantially fewer calories than they expend during prolonged events, often resulting in marked negative energy balance [20,22,24,30,31,50]. While a degree of energy deficit is inevitable during ultra-endurance competition, concerns arise when athletes repeatedly fail to restore adequate energy availability during recovery periods. Such patterns may contribute to chronic LEA and increase susceptibility to REDs-related consequences [47,49-51]. 

Female ultra-endurance athletes appear particularly vulnerable to menstrual disturbances associated with chronic LEA [24,35,36,52]. Functional hypothalamic amenorrhoea remains an important clinical manifestation because of its association with impaired bone health and increased injury risk [53]. Similarly, studies across endurance populations consistently demonstrate that menstrual dysfunction, low body mass, previous stress fracture, and inadequate energy availability are among the strongest predictors of future bone stress injury [13,41,43,48].

Challenges in Estimating the True Burden of REDs

A major challenge in understanding the epidemiology of REDs is that many affected athletes remain undiagnosed. Symptoms such as fatigue, recurrent illness, reduced libido, menstrual dysfunction, impaired recovery, and declining performance often develop gradually and may be attributed to training rather than underlying LEA [13,43]. Furthermore, endurance sport culture frequently values resilience, perseverance, and tolerance of discomfort, characteristics that may inadvertently normalise physiological dysfunction and discourage help-seeking behaviour [4,54]. Consequently, current prevalence estimates likely underestimate the true burden of REDs within ultra-endurance populations. Many athletes may continue training and competing despite substantial physiological compromise, only presenting for medical assessment following injury, prolonged underperformance, or significant health deterioration [4,54].

Despite increasing recognition of REDs across endurance sport, substantial knowledge gaps remain. Direct evidence from ultra-trail running populations remains limited, and there is currently no large prospective cohort study establishing the prevalence of REDs among ultra-trail athletes [48]. Similarly, the prevalence of LEA in recreational ultra-trail runners, sex-specific manifestations of REDs, long-term endocrine consequences of repeated ultra-endurance participation, relationships with race completion and injury risk, and the effectiveness of current screening tools all remain poorly understood [48]. Addressing these gaps will be essential for developing sport-specific prevention, screening, and management strategies as participation in ultra-trail running continues to expand internationally.

Why ultra-trail running creates a unique REDs environment

Ultra-trail running presents a unique context for the development of REDs because athletes are exposed to multiple physiological and behavioural risk factors simultaneously [4,54]. Unlike many traditional endurance sports, ultra-trail participation combines prolonged training volumes, substantial race-related energy expenditure, gastrointestinal limitations, sleep disruption, environmental stress, and cultural pressures surrounding body composition and performance [3,15,20,21,24]. Collectively, these factors create an environment in which maintaining adequate energy availability becomes particularly challenging.

Extreme Energy Demands and Chronic Energy Deficit

The most obvious contributor to REDs risk in ultra-trail running is the extraordinary energetic demand associated with both training and competition. Competitive athletes frequently undertake weekly training volumes exceeding 80 to 150 km, while elite runners may accumulate 15 to 25 hours of training during peak preparation phases [16,24,47]. Such workloads can generate daily energy expenditures well above 3,500 to 5,000 kcal and may increase substantially during intensive training blocks [16,20,22,47]. Importantly, LEA in ultra-trail athletes often develops unintentionally. Rather than deliberate dietary restriction, many runners simply struggle to match energy intake to expenditure over prolonged periods [21,22,49]. Small daily deficits may accumulate progressively, resulting in chronic LEA despite apparently reasonable nutritional habits [16,31,47,48]. The challenge becomes even greater during competition, where energy expenditure frequently exceeds the body's capacity for replacement, regardless of nutritional planning [21,31,49]. Ultramarathon studies consistently demonstrate substantial caloric deficits during races, particularly during multi-day events in which sleep deprivation and gastrointestinal symptoms further impair intake [21,29,55]. Prolonged exercise may also suppress appetite through alterations in appetite-regulating hormones, including ghrelin, peptide YY, and glucagon-like peptide-1 [56]. Consequently, athletes may struggle to adequately replenish energy stores following demanding training sessions or competitions, promoting cumulative energy deficiency over time [30,50,55,57].

Gastrointestinal and Nutritional Challenges

Gastrointestinal symptoms represent a distinctive feature of ultra-endurance participation and may further compromise energy availability. Nausea, bloating, abdominal discomfort, reflux, diarrhoea, and appetite suppression are frequently reported during both training and competition [21,29,56]. These symptoms arise through a combination of intestinal hypoperfusion, mechanical stress associated with running, dehydration, environmental exposure, and nutritional factors [21,56,58,59]. The practical consequence is that athletes often consume substantially less energy than intended despite recognising the importance of fuelling [16,30,47,60]. In severe cases, prolonged periods of inadequate intake may occur during races, contributing to significant acute energy deficits. Because many ultra-trail events take place in remote environments with limited nutritional options, logistical barriers may compound these physiological challenges [21,30,59,61].

Additional Physiological Stressors

Ultra-trail athletes are also exposed to stressors that may amplify the consequences of LEA. Sleep deprivation is common during races exceeding 100 miles and may be severe during multi-day competitions, with some athletes having as little as an hour's sleep during a 56-hour effort [19,61-63]. Sleep plays a critical role in endocrine regulation, glycogen restoration, tissue repair, immune function, and psychological recovery [19,61-63]. Consequently, sleep restriction may potentiate many of the physiological pathways implicated in REDs, including elevated cortisol concentrations, impaired glucose regulation, and reduced anabolic hormone production [19,56,61,62]. Environmental conditions further increase physiological demands. Heat, cold, altitude, humidity, and variable weather can all influence substrate utilisation, fluid balance, gastrointestinal function, and total energy expenditure [21,29,30,58,59]. These factors often coexist during mountain ultra-trail events, creating an additional challenge to maintaining adequate energy availability.

Sociocultural Contributors

Beyond physiological factors, cultural influences may also contribute to the risk of developing REDs. Although ultra-trail running lacks the overt aesthetic emphasis observed in some sports, lower body mass is often perceived as advantageous for climbing efficiency and running economy [64,65]. This perception may encourage intentional or unintentional energy restriction through increased training volume, dietary manipulation, intermittent fasting, or pursuit of a specific race weight [65,66]. Social media may further reinforce these beliefs by highlighting elite physiques, training metrics, and weight-performance narratives [65,66].

Endurance sport culture itself may also delay recognition of REDs. Resilience, perseverance, and tolerance of discomfort are highly valued characteristics within ultra-trail communities [4,54,66]. While these attributes contribute positively to performance, they may also encourage athletes to normalise fatigue, recurrent injury, menstrual dysfunction, and inadequate recovery [3,48,54,66]. As a result, physiological warning signs may go unrecognised until substantial health or performance consequences emerge.

Ultra-trail running combines multiple established REDs risk factors within a single sporting environment. Extreme energetic demands, gastrointestinal limitations, sleep disruption, environmental stressors, body composition pressures, and cultural influences collectively create conditions highly conducive to the development of LEA. These unique characteristics provide a strong theoretical basis for considering ultra-trail runners a population at elevated risk of REDs and support the need for increased awareness, targeted screening, and sport-specific research.

Clinical and performance consequences of REDs in ultra-trail running

The consequences of REDs extend beyond isolated physiological abnormalities and have important implications for both athletic performance and long-term health. In ultra-trail running, where success depends upon the interaction of endurance capacity, musculoskeletal resilience, nutritional adequacy, recovery, and psychological robustness, chronic LEA may impair multiple determinants of performance simultaneously [11,19,46,51,67]. Importantly, these effects often develop gradually and may initially be interpreted as inadequate training, ageing, or the expected consequences of participation in a demanding sport.

One of the earliest manifestations of LEA is impaired adaptation to training [11,32,33]. Endurance performance relies upon repeated cycles of physiological stress and recovery that drive mitochondrial biogenesis, capillary development, neuromuscular adaptation, and improvements in substrate utilisation [3]. These adaptive processes require adequate energy availability. When energy intake becomes insufficient, the body prioritises essential physiological functions over performance optimisation, resulting in reduced training responsiveness despite an ongoing workload [3,54]. Athletes frequently describe stagnating performance, reduced ability to complete key sessions, and a perception of declining fitness despite increasing training effort [11,12,46]. Over time, this mismatch may contribute to a cycle in which worsening performance prompts further increases in training volume, thereby exacerbating the underlying energy deficit.

Recovery impairment represents another important consequence of REDs. Restoration of glycogen stores, tissue repair, protein synthesis, and endocrine recovery all require sufficient energy intake [3,32,43,55]. Chronic LEA may therefore prolong recovery following both training and competition. Athletes commonly report persistent fatigue, prolonged muscle soreness, reduced tolerance to training load, and difficulty maintaining consistency within training programmes [11,12,46,67]. Such effects may be particularly important in ultra-trail running, where high-volume training blocks, back-to-back long runs, and frequent competition place substantial demands upon recovery systems [3].

Musculoskeletal injury is among the most clinically significant consequences of REDs. The strongest evidence relates to bone health, where chronic LEA disrupts normal bone remodelling through endocrine adaptations that favour energy conservation at the expense of skeletal maintenance. Reduced bone formation and impaired bone mineral density increase susceptibility to bone stress injuries, including stress reactions and stress fractures [13,32,43]. Female athletes with menstrual dysfunction demonstrate particularly high injury risk, although similar associations with the hypogonadal axis are increasingly recognised in male athletes [36,38,41,45]. Within ultra-trail running, common sites of bone stress injury include the tibia, metatarsals, fibula, pelvis, sacrum, and femoral neck, reflecting the repetitive loading associated with prolonged running on variable terrain [13,43]. The consequences extend beyond temporary interruption of training, as prolonged rehabilitation, recurrent injury, and incomplete recovery may substantially affect long-term athletic development.

Beyond skeletal injury, REDs may also contribute to tendon pathology and impaired connective tissue health [43,68]. Tendon adaptation requires adequate nutritional support and collagen synthesis, processes that may be compromised during chronic energy deficiency [3,15]. Although direct evidence remains limited, athletes with LEA frequently report persistent tendinopathies, delayed healing, and recurrent overuse injuries [68]. Clinicians should therefore consider REDs in athletes presenting with musculoskeletal complaints that appear disproportionate to training load or demonstrate poor response to conventional management.

Endocrine dysfunction represents a hallmark feature of REDs and underpins many of its clinical manifestations [35,36,38,41,45]. Chronic LEA is associated with reductions in reproductive hormones, insulin-like growth factor-1, leptin, and triiodothyronine, alongside elevations in stress-related hormonal pathways [34,35,38,45,53]. In female athletes, menstrual dysfunction ranging from oligomenorrhoea to functional hypothalamic amenorrhoea remains one of the most important clinical indicators of physiological compromise [53]. In male athletes, manifestations may include reduced testosterone concentrations, diminished libido, impaired recovery, and unexplained performance decline [37-39,45]. While some endocrine adaptations may represent attempts to conserve energy, prolonged disruption may adversely affect health, recovery, and athletic performance.

Immune function may also be compromised. Athletes experiencing REDs appear more susceptible to recurrent illness, particularly upper respiratory tract infections. Reduced energy availability, endocrine disruption, elevated cortisol concentrations, and potential micronutrient deficiencies may collectively impair immune surveillance and recovery [68,69]. Given that prolonged endurance exercise itself imposes immunological stress, LEA may further increase vulnerability during periods of intensive training or competition.

The impact of REDs on race-day performance is likely multifactorial. Reduced glycogen availability, impaired recovery, cumulative fatigue, endocrine dysfunction, and increased injury burden may collectively diminish physiological reserve entering competition [3,25,26,69,70]. Emerging evidence also suggests that chronic energy deficiency may influence mood, concentration, decision-making, and perceived exertion [4,54]. Such effects may be particularly relevant in ultra-trail running, where pacing strategy, nutritional decision-making, navigation, and cognitive resilience become increasingly important as race duration increases [71,72]. Although direct evidence linking REDs to race outcomes remains limited, it is plausible that chronic LEA contributes to underperformance and may increase the likelihood of race withdrawal or failure to finish [28,73].

Perhaps the most important consequence of REDs is the impact on long-term athlete health and longevity [48]. Ultra-trail running is unusual in that many athletes remain competitive well into middle age and beyond [1,2,48]. Sustained participation, therefore, depends not only upon short-term performance optimisation but also upon preserving skeletal health, endocrine function, cardiac health, and musculoskeletal resilience over many years [26-28,43,48,68]. Persistent LEA may increase lifetime fracture risk, impair attainment or maintenance of bone mineral density, disrupt reproductive health, and contribute to recurrent injury patterns that limit long-term participation [26,48,68]. While many physiological disturbances improve following restoration of adequate energy availability, delayed recognition may result in consequences that are only partially reversible. 

Collectively, these findings highlight that REDs should not be viewed solely as a nutritional issue or a cause of declining performance. Rather, it represents a multisystem condition with important implications for athlete health, injury risk, recovery, and sustainable participation in sport. For ultra-trail runners, whose success depends upon long-term consistency and resilience, early recognition and management of LEA may be critical for both competitive performance and lifelong health.

Screening, diagnosis, and management of REDs in ultra-trail running

Recognition of REDs remains challenging because there is no single diagnostic test capable of confirming or excluding the condition [7,8,13,40]. Instead, diagnosis relies upon integration of clinical history, physical examination, physiological assessment, laboratory investigations, and consideration of sport-specific risk factors [7,24,40]. This challenge is particularly relevant in ultra-trail running, where symptoms commonly associated with REDs, including fatigue, weight loss, musculoskeletal discomfort, disrupted sleep, and prolonged recovery, are often considered normal consequences of participation [3]. Consequently, clinicians caring for ultra-endurance athletes must maintain a high index of suspicion and adopt a proactive approach to screening [64]. Early identification is especially important because many physiological consequences of REDs develop gradually and may become increasingly difficult to reverse if intervention is delayed.

Challenges in Identifying REDs in Ultra-Trail Athletes

Several characteristics of ultra-trail running complicate diagnosis. First, many athletes maintain impressive levels of performance despite substantial physiological compromise [4,54,64,71]. Successful race completion or high training volume should not therefore be interpreted as evidence against REDs. Second, symptoms often develop insidiously. Athletes may adapt to progressively worsening fatigue, declining recovery, recurrent illness, or menstrual disturbance without recognising these changes as abnormal [68]. In many cases, presentation occurs only after development of a stress fracture, prolonged underperformance, or significant endocrine dysfunction [24]. Third, BMI may remain within the normal range despite substantial LEA. Unlike severe eating disorders, REDs frequently occur in athletes who appear outwardly healthy and may not exhibit obvious weight loss [3,24,28]. Reliance upon body composition alone is therefore insufficient. Finally, many ultra-trail runners demonstrate personality traits, including resilience, perseverance, and high achievement orientation. While advantageous for performance, these characteristics may delay help-seeking and contribute to normalisation of physiological symptoms [4,54,66,71,72].

Clinical Assessment of REDs in Ultra-Trail Athletes

A comprehensive clinical assessment remains the cornerstone of REDs identification and diagnosis. Because no single symptom, physical finding, laboratory marker, or screening tool is sufficiently sensitive or specific to establish the diagnosis independently, clinicians must integrate information from multiple domains to determine the likelihood and severity of LEA and its physiological consequences [3,24,54].

The clinical history is particularly important and should explore training, nutrition, reproductive health, injury history, and broader indicators of recovery and performance. Training history should include an assessment of weekly training volume, the frequency and duration of long runs, recent changes in training load, race participation patterns, cross-training activities, and recovery practices. Particular attention should be paid to abrupt increases in workload, repeated competition without adequate recovery, and prolonged periods of high-volume training, all of which may increase susceptibility to LEA and subsequent REDs-related complications [64].

Given the absence of validated ultra-trail-specific screening pathways, a structured clinical assessment framework may assist clinicians in identifying athletes at risk of REDs. Assessment should begin with a detailed exploration of training load, nutritional practices, and symptom burden. Key questions include: (1) Has training volume or intensity increased substantially in recent weeks or months? (2) Does the athlete regularly skip meals, delay post-exercise nutrition, utilise intermittent fasting strategies, or restrict specific food groups? (3) Are symptoms such as persistent fatigue, recurrent illness, impaired recovery, declining performance, menstrual disturbance, reduced libido, or recurrent musculoskeletal injuries present? (4) Do symptoms improve during periods of reduced training load, injury, or increased nutritional intake? (5) Is there evidence of functional impairment affecting training consistency, competition performance, occupational activities, or psychological wellbeing?

Nutritional assessment should extend beyond simple estimates of caloric intake. Fat-free mass should be reassessed periodically, particularly during periods of changing training load, injury, or body composition change, as fluctuations may substantially influence calculated energy availability. Clinicians should evaluate daily meal frequency, timing of nutritional intake relative to training sessions, carbohydrate availability, dietary restrictions, use of intermittent fasting strategies, gastrointestinal symptoms, and race-day fuelling practices [33,47,49,50,57,60]. Importantly, many athletes who develop REDs do not intentionally restrict energy intake and may genuinely believe that they are consuming sufficient nutrition to support their training demands [60]. Consequently, a detailed exploration of fuelling behaviours is often required to identify inadvertent under-fuelling.

Athletes reporting persistent symptoms, evidence of endocrine dysfunction, recurrent bone stress injury, prolonged performance decline, or significant functional impairment should undergo further multidisciplinary evaluation, including sports medicine, sports dietetics, and, where appropriate, endocrinology assessment. While this framework has not been formally validated, it provides a pragmatic approach to identifying athletes who may benefit from further investigation and early intervention.

Among female athletes, menstrual history provides valuable insight into physiological status and should form a routine component of REDs assessment. Clinicians should enquire about age at menarche, cycle regularity, recent changes in cycle length, episodes of oligomenorrhoea or amenorrhoea, and the use of hormonal contraception. Menstrual dysfunction should never be regarded as a normal adaptation to endurance training and may represent an important clinical marker of prolonged LEA [35,36,41]. In male athletes, assessment of reproductive health may include discussion of libido, erectile function, and other symptoms suggestive of hypogonadism, particularly when fatigue, impaired recovery, or unexplained declines in performance are present [37-39,45].

A detailed injury and illness history is equally important. Recurrent stress fractures, persistent tendinopathies, frequent infections, and prolonged recovery following training or competition should raise suspicion of underlying LEA [68]. The combination of recurrent injury and progressive performance decline is particularly concerning and warrants comprehensive investigation for REDs and related physiological dysfunction. Several screening instruments have been developed to assist with the identification of athletes at risk. The LEAF-Q remains one of the most widely utilised screening tools in female athletic populations [40]. The questionnaire assesses menstrual function, gastrointestinal symptoms, and injury history, with higher scores associated with an increased likelihood of LEA. More recently, the IOC REDs CAT2 provides a structured framework for both clinical assessment and return-to-sport decision-making [8]. The REDs CAT2 incorporates clinical symptoms, physiological findings, psychological factors, performance indicators, and relevant medical history to stratify athletes according to risk. Importantly, the tool emphasises that REDs exists along a spectrum of severity rather than representing a simple binary diagnosis [8]. Although research into novel screening approaches continues, including sport-specific questionnaires and digital monitoring systems, no validated screening instrument currently exists specifically for ultra-trail runners [24]. This represents an important limitation given the distinctive physiological demands and cultural characteristics of ultra-endurance participation.

Physical examination findings in athletes with REDs are often subtle and may be entirely absent despite significant physiological disruption [11,24,32]. Potential clinical indicators include low body mass, recent weight loss, orthostatic hypotension, bradycardia, delayed recovery from injury, and reduced muscle mass [11,24,32]. However, many athletes with REDs demonstrate entirely normal physical examinations. Consequently, the absence of obvious clinical findings should not reassure clinicians when historical features strongly suggest LEA.

Laboratory investigations may provide supportive evidence but should always be interpreted within the broader clinical context. Initial investigations commonly include a full blood count, urea and electrolytes, liver function tests, bone profile, vitamin D, ferritin, vitamin B12, and folate concentrations [11,24,74]. Endocrine assessment may also be indicated. In female athletes, measurement of luteinising hormone, follicle-stimulating hormone, oestradiol, prolactin, and thyroid function can help identify reproductive and metabolic disturbances [35,74]. In male athletes, testosterone, luteinising hormone, follicle-stimulating hormone, and sex hormone-binding globulin may provide insight into hypothalamic-pituitary-gonadal axis suppression [38,39,45,74,75]. Assessment of cortisol, insulin-like growth factor-1, and thyroid hormones may be appropriate in both sexes [74]. Several studies have demonstrated reductions in T3 concentrations among athletes with chronic LEA, suggesting a potentially useful biomarker when interpreted alongside other clinical findings [74]. Nevertheless, no laboratory parameter currently possesses sufficient sensitivity or specificity to independently diagnose REDs.

Given the strong association between REDs and impaired skeletal health, evaluation of bone status is frequently warranted [13,32,43]. Dual-energy X-ray absorptiometry (DXA) remains the gold standard for assessment of bone mineral density and is particularly valuable in athletes with amenorrhoea lasting longer than six months, recurrent stress fractures, suspected osteoporosis, or prolonged periods of LEA [13,32,43]. In addition to bone mineral density measurements, DXA provides useful information regarding body composition, including fat-free mass and regional lean tissue distribution. Athletes presenting with focal bone pain should undergo prompt imaging to exclude stress injury. The MRI remains the preferred modality for early diagnosis because it can identify bone stress reactions before progression to overt fracture [32,43]. Early detection is particularly important in ultra-trail athletes, as timely intervention may substantially reduce recovery time, minimise training disruption, and prevent progression to more serious skeletal injury.

Principles of Management and Prevention of REDs in Ultra-Trail Running

The primary objective of the management of REDs is the restoration of adequate energy availability and the reversal of the physiological adaptations that arise in response to chronic energy deficiency. Successful treatment requires addressing the underlying mismatch between energy intake and exercise expenditure rather than focusing exclusively on individual manifestations such as menstrual dysfunction, impaired bone health, endocrine abnormalities, or recurrent injury [5,33,42,46,74,76]. Consequently, management strategies should prioritise correction of the energy deficit while simultaneously supporting recovery, health, and athletic performance.

For most athletes, increasing energy intake represents the cornerstone of treatment [11,12,74,76]. Nutritional interventions should focus on improving overall energy availability through increased meal frequency, incorporation of structured snacks, optimisation of post-exercise nutrition, enhancement of carbohydrate availability, and greater energy density of meals [11,16,20,33,55]. Importantly, nutritional recommendations should be individualised according to training demands, body composition goals, gastrointestinal tolerance, and the practical realities of ultra-endurance participation [16,20,29,47,57]. Athletes frequently underestimate the energy requirements associated with high-volume training, particularly during periods of intensified preparation, and education regarding appropriate fuelling strategies therefore forms a critical component of management. In some circumstances, increasing energy intake alone may be insufficient to restore energy balance. Temporary modification of training load may be necessary, particularly in the presence of stress fractures, severe fatigue, significant endocrine dysfunction, persistent illness, or impaired recovery [64]. Reductions in training volume, intensity, or competition frequency can help reduce overall energy expenditure and facilitate physiological restoration. Such interventions are often psychologically challenging for ultra-trail athletes, many of whom derive a substantial component of their identity, social network, and psychological wellbeing from training and competition [54,65,66]. Clear communication regarding the rationale for temporary training modification is therefore essential, with emphasis placed upon the long-term benefits for both health and future athletic performance.

Optimal management frequently requires a multidisciplinary approach involving collaboration between multiple healthcare professionals [24,74]. Depending upon the severity and manifestations of REDs, the multidisciplinary team may include a sports physician, sports dietitian, physiotherapist, endocrinologist, psychologist, and strength and conditioning coach [24,64,74]. This collaborative model allows simultaneous management of nutritional deficits, injury rehabilitation, endocrine disturbances, psychological factors, and return-to-sport planning [46]. Psychological support may be particularly valuable when disordered eating behaviours, body image concerns, perfectionistic tendencies, or exercise dependence contribute to the development or persistence of LEA [4,24,41,54,66]. Importantly, management should remain athlete-centred and prioritise long-term health outcomes rather than focusing solely on restoring competition eligibility.

Although effective treatment is essential, prevention remains the preferred strategy. Given the prolonged training volumes and substantial energy expenditures characteristic of ultra-trail running, proactive measures aimed at maintaining adequate energy availability may substantially reduce the risk of REDs. Education represents one of the most important preventive interventions. Athletes should understand the concept of energy availability, recognise the early signs and symptoms of REDs, appreciate the importance of menstrual and reproductive health, and understand the potential consequences of chronic under-fuelling on performance, recovery, and long-term health [64].

Contemporary sports nutrition recommendations increasingly advocate a 'fuel for the work required' approach, whereby nutritional intake is matched dynamically to training demands rather than maintaining chronically restricted energy intake [16,20,47,57]. This concept is particularly relevant in ultra-trail running, where training load often fluctuates substantially throughout the season. Periods of heightened risk include high-volume training blocks, back-to-back long runs, multi-day stage races, and intensive recovery phases following competition, all of which may significantly increase energy requirements [16,20,47].

Regular monitoring of recovery and health indicators may facilitate earlier identification of athletes developing LEA before more serious complications arise. Athletes should be encouraged to monitor menstrual function, perceived recovery quality, injury occurrence, illness frequency, mood state, sleep quality, and training tolerance [24,68]. Changes in these variables may provide important early warning signs and prompt timely intervention before progression to more severe manifestations of REDs. Coaches also play a critical role in prevention. As primary influencers of training practices and performance culture, coaches are often well positioned to identify behavioural or physiological changes that may indicate inadequate fuelling. Improved awareness of REDs among coaches may facilitate earlier recognition of at-risk athletes while helping to challenge potentially harmful beliefs regarding body composition, weight loss, and excessive training volume [64]. Creating training environments that prioritise health, recovery, and sustainable performance may be particularly important within ultra-endurance communities, where perseverance through discomfort is often culturally valued.

**Figure 1:**
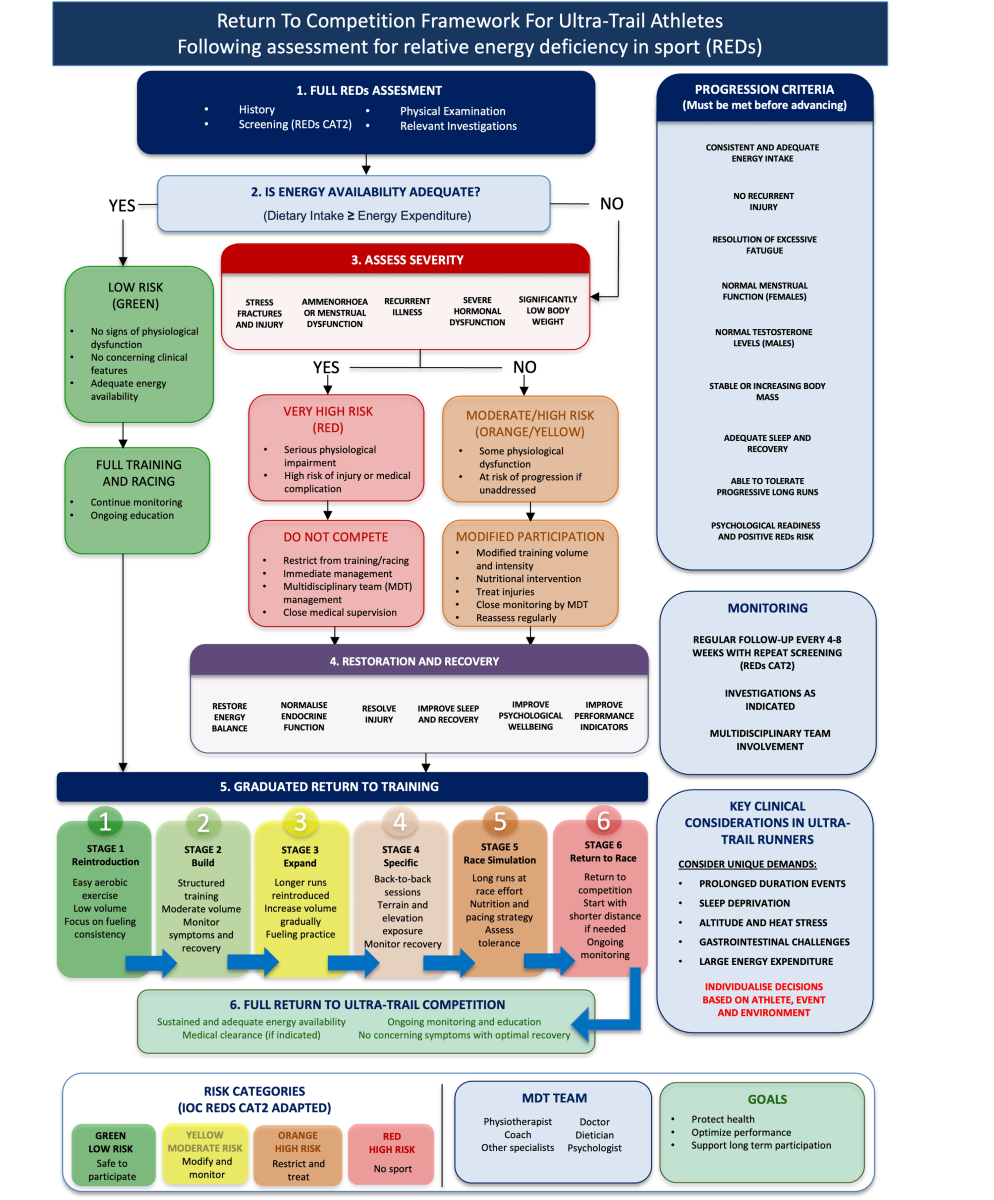
Proposed return-to-competition framework for ultra-trail athletes following REDs assessment Athletes are stratified according to energy availability, physiological dysfunction, injury burden, and clinical risk. Progression from modified participation to full competition should occur only following restoration of adequate energy availability, clinical improvement, and resolution of significant health consequences. This flowchart (created by the author using Microsoft PowerPoint without generative AI tools such as Microsoft Copilot) represents a schematic adapted from principles outlined by the IOC REDs CAT2, with additional considerations specific to ultra-trail running [8]. REDs: Relative energy deficiency in sport, IOC: International Olympic Committee, CAT2: Clinical assessment tool version 2

Return-to-sport decisions following REDs should be individualised and guided by structured risk stratification frameworks such as the IOC REDs CAT2 [8]. Assessment should consider restoration of adequate energy availability, improvement in hormonal and metabolic function, resolution of injury, adherence to nutritional recommendations, and psychological readiness to return to training and competition. Progression should occur gradually and be supported by ongoing monitoring to ensure that physiological recovery is maintained. Premature return to unrestricted training may increase the risk of recurrent LEA, delayed recovery, and further health complications, ultimately compromising both athletic performance and long-term wellbeing. A proposed pathway for return to competition for ultra-trail athletes is depicted in Figure 1.

The diagnosis of REDs requires a comprehensive and systematic approach integrating clinical history, screening tools, laboratory investigations, and assessment of bone health. Ultra-trail athletes present unique challenges because many symptoms overlap with expected consequences of endurance training. Effective management centres upon restoration of adequate energy availability through nutritional intervention, training modification, and multidisciplinary support. Given the potential consequences for both performance and long-term health, prevention and early identification should remain priorities within the ultra-trail community.

Current controversies and future directions

Despite growing recognition of REDs within sports medicine, significant uncertainty remains regarding its prevalence, diagnosis, and clinical significance in ultra-trail running. Much of the existing evidence is derived from female athletes, collegiate populations, and sports traditionally associated with body composition pressures. Consequently, extrapolation of these findings to ultra-endurance athletes should be undertaken cautiously.

Under-Diagnosis Versus Over-Diagnosis

One of the central controversies surrounding concerns of REDs is whether the condition remains under-recognised or is increasingly being overdiagnosed [11,24,32]. Increased awareness has undoubtedly improved identification of athletes with menstrual dysfunction, recurrent injury, endocrine disturbance, and chronic fatigue [25,41,60,74]. However, some authors have questioned whether expanding the frameworks of REDs risks pathologising physiological adaptations that may occur in highly trained endurance athletes. This debate is particularly relevant in ultra-trail running, where athletes frequently experience transient reductions in energy availability, altered hormonal profiles, bradycardia, and substantial fluctuations in body mass [3,11,15,25,28]. Distinguishing adaptive responses from clinically significant dysfunction remains challenging. Current evidence suggests that REDs should be considered when inadequate energy availability persists sufficiently to impair health, recovery, or performance rather than in response to temporary physiological changes alone.

Is Negative Energy Balance During Competition Pathological?

A related question concerns the interpretation of race-induced energy deficits. During ultramarathons and multi-day events, total energy expenditure frequently exceeds the body's capacity for replacement regardless of nutritional planning [16,33,47,50,60]. Consequently, a substantial negative energy balance should generally be regarded as a normal physiological consequence of participation rather than evidence of pathology. The critical issue appears to be recovery. Athletes who repeatedly experience large energy deficits without adequate restoration of energy availability may be at greater risk of developing chronic LEA and REDs. Future research should therefore focus not only on race-day energy balance but also on post-race recovery and cumulative energetic stress across training cycles.

Diagnostic Limitations

Although the IOC REDs framework represents a major advance in athlete health, important diagnostic limitations remain [7,8,77]. Direct measurement of energy availability is impractical in most clinical settings, while many clinical manifestations of REDs lack specificity [42]. Fatigue, reduced performance, recurrent illness, and endocrine disturbance may result from numerous causes, including overtraining, sleep deprivation, iron deficiency, psychological stress, or underlying medical conditions [19,28,63,66,74]. Furthermore, proposed thresholds for clinically significant LEA are derived primarily from studies involving female athletes and may not be directly applicable to male athletes, masters competitors, or ultra-endurance populations. These limitations reinforce the importance of comprehensive clinical assessment rather than reliance upon any individual marker or screening tool.

Evidence Specific to Ultra-Trail Running

Despite the rapid growth of ultra-trail running, direct evidence examining REDs within this population remains limited [1,2,24]. Existing concerns are supported largely by indirect observations, including substantial race-related energy deficits, high rates of gastrointestinal symptoms, prolonged recovery requirements, and exposure to multiple physiological stressors. However, no large prospective cohort studies have established the prevalence of REDs among ultra-trail runners, and no validated ultra-endurance-specific screening instrument currently exists. As a result, many current recommendations remain extrapolated from broader endurance-sport literature.

Future Research Directions

Despite increasing recognition of REDs in endurance sport, important evidence gaps remain within ultra-trail running. The true prevalence of LEA and REDs among ultra-trail athletes remains unknown, as most available data are derived from small cross-sectional studies or extrapolated from other endurance populations. Large prospective cohort studies involving both elite and recreational athletes are therefore required to establish prevalence estimates and identify sport-specific risk factors.

Current screening approaches also require further refinement. Existing tools such as the LEAF-Q and REDs CAT2 were not developed specifically for ultra-endurance athletes and may not fully account for the unique physiological demands of ultra-trail running, including prolonged energy deficits, gastrointestinal dysfunction, sleep deprivation, and environmental stress [8,40]. Development and validation of ultra-endurance-specific screening instruments should therefore represent a research priority. Greater attention should also be directed towards male athletes, who remain under-represented in the REDs literature. Improved understanding of male-specific manifestations, endocrine adaptations, and diagnostic thresholds is required to facilitate earlier recognition and intervention. The relationship between REDs and ultra-trail-specific outcomes warrants further investigation. Future studies should examine associations between LEA and race completion, injury incidence, illness frequency, recovery, pacing strategies, and long-term athletic performance. Such work may help clarify whether REDs contribute to increased risk of race withdrawal and underperformance in ultra-endurance competition.

Finally, longitudinal and interventional research is urgently needed. Long-term studies examining skeletal health, endocrine recovery, reproductive function, and healthy ageing would improve understanding of the lifelong consequences of REDs. Similarly, trials evaluating nutritional education, coach-focused interventions, and multidisciplinary prevention programmes are required to establish evidence-based strategies for reducing REDs risk within the growing ultra-trail community.

## Conclusions

Ultra-trail running has evolved into a globally recognised endurance discipline characterised by prolonged exercise duration, substantial energy expenditure, environmental stress, technical terrain, and increasingly competitive participation. These unique demands create a physiological environment in which maintaining adequate energy availability can be exceptionally challenging.

Relative energy deficiency in sport represents a significant but likely under-recognised threat to both performance and long-term athlete health within the ultra-distance trail running population. Extreme training volumes, race-induced energy deficits, gastrointestinal dysfunction, appetite suppression, sleep disruption, and cultural beliefs surrounding body composition collectively increase susceptibility to chronic LEA. Consequences extend beyond impaired performance and include endocrine dysfunction, impaired bone health, recurrent illness, delayed recovery, and increased injury risk, something that is already being seen across elite competitions through increasing did-not-finish (DNF) rates.

Although the physiological demands of ultra-trail running and the health consequences of REDs have been independently described, a significant gap remains in understanding the prevalence, presentation, and long-term consequences of REDs in ultra-trail athletes specifically. Current knowledge is largely extrapolated from studies involving road runners, collegiate athletes, and other endurance populations, limiting the applicability of existing evidence to the unique demands of ultra-trail running. Furthermore, the interaction between prolonged race-induced energy deficits, sleep deprivation, environmental stress, and chronic LEA remains poorly characterised. Prospective studies evaluating energy availability, endocrine function, bone health, injury incidence, and performance outcomes in ultra-trail cohorts are therefore needed to establish sport-specific screening, prevention, and management strategies.

Clinicians, coaches, and athletes should recognise that persistent fatigue, menstrual dysfunction, recurrent injury, prolonged recovery, and declining performance are not inevitable consequences of endurance training but may represent important indicators of inadequate energy availability. As participation in ultra-trail running continues to expand, greater emphasis on education, early identification, and evidence-based management will be essential to support both sustainable performance and lifelong athlete health.
